# Altered regulation of Prox1-gene-expression in liver tumors

**DOI:** 10.1186/1471-2407-8-92

**Published:** 2008-04-09

**Authors:** Jozsef Dudas, Tümen Mansuroglu, Federico Moriconi, Florian Haller, Joerg Wilting, Thomas Lorf, Laszlo Füzesi, Giuliano Ramadori

**Affiliations:** 1 Department of Internal Medicine, Section of Gastroenterology and Endocrinology, Georg-August-University Goettingen, Robert-Koch-Strasse 40, 37075 Goettingen, Germany; 2 Department of Gastroenteropathology, Georg-August University Goettingen, Robert-Koch-Strasse 40, 37075 Göttingen, Germany; 3 Department of Anatomy and Cell Biology, Georg-August-University Goettingen, Kreuzbergring 36, 37075 Goettingen, Germany; 4 Department of General Surgery, Georg-August-University Goettingen, Robert-Koch-Strasse 40, 37075 Goettingen, Germany

## Abstract

**Background:**

Prospero-related homeobox 1 (Prox1) transcription factor was described as a tumor-suppressor gene in liver tumors. In contrast, Prox1 knock out in murine embryos drastically reduces proliferation of hepatoblasts.

**Methods:**

We have studied the expression of Prox1 in normal liver, liver cirrhosis and peritumoral liver samples in comparison to hepatocellular (HCC) and cholangiocellular carcinoma (CCC) at mRNA, protein and functional levels.

**Results:**

Prox1 was found in hepatocytes of normal liver, while normal bile duct epithelial cells were negative. However, Prox1^+ ^cells, which co-expressed biliary epithelial makers and showed ductular morphology, could be detected within fibrotic septa of cirrhotic livers, and in both HCC and CCC. Two Prox1 mRNA isoforms (2.9 kb and 7.9 kb) were identified with a prevalence of the longer isoform in several HCC samples and the shorter in most CCC samples. Evidence was provided that Myc-associated zinc finger protein (MAZ) might significantly contribute to the gene expression of Prox1 in HCC, while neo-expression of Prox1 in CCC remains to be resolved. A point mutation in the prospero domain of Prox1 was found in one HCC sample.

**Conclusion:**

Our study shows dysregulation of Prox1 in liver cirrhosis, HCC and CCC, such as neo-expression in cells with biliary epithelial phenotype in liver cirrhosis, and in CCC. Altered Prox1 mRNA expression is partly regulated by MAZ, and mutation of the prospero domain in HCC indicates an involvement for Prox1 during tumor progression.

## Background

Homeobox genes encode transcription factors that control cell differentiation and play essential roles during embryonic development [1,2]. The homeobox genes Hlx [3], Hex [4] and the prospero-related homeobox 1 (Prox1) [5] are involved in the development of the liver bud. Prox1 belongs to the earliest markers of mammalian foregut endoderm cells, where its expression is confined to a short segment that gives rise both to the liver and pancreas, remaining expressed during the adulthood [6,7]. In *Prox1*-deficient mice, the hepatoblasts fail to migrate into the neighboring mesenchyme [8]. Prox1-null mice die around embryonic day (ED) 14.5 and show a 70% reduction of liver size [8]. Besides albumin and α-fetoprotein (AFP), Prox1 is an early marker of hepatoblasts [9]. In our previous study it was shown that Prox1 remains a stabile marker of hepatocytes in normal adult, injured and regenerating liver, whereas it is not detectable in biliary epithelial cells [10]. Prox1 was found to regulate metabolic function in hepatocytes, and to be involved in the regulation of other transcription factors [11].

A previous study showed various levels of *prox1 *mRNA expression in HCC cell lines and tumor samples [12], and found that, patients with higher expression had much more favorable prognosis than those with lower expression. On the other hand, there was no statistically significant difference in the disease-free survival rates between high and low expression of prox1.

A down-regulation of *prox1*-gene-expression was reported in HCC and CCC liver samples *compared to normal liver *[12,13]. It was supposed that downregulation of *prox1*-gene-expression was induced by the methylation of the Prox1-promoter [13]. Additionally, so far, no mutations were found in the coding region of Prox1 in HCCs [12]. However, comparing the gene expression pattern or *prox1*, a decreased expression could be found in the average of HCC and CCC liver samples, whereas, a stable high expression level persists in HCC cell lines, and in the MZ-Cha2 biliary adenocarcinoma cell line.

Aim of the current study was to investigate the regulatory changes of Prox1-gene-expression in liver cirrhosis, in hepatocellular and cholangiocellular carcinomas. While, as we recently reported, no Prox1-expression was found in biliary epithelial cells of the normal liver [10], it was newly expressed in ductular cells showing biliary epithelial cell makers within the fibrotic septa of cirrhotic livers, in HCC and CCC. In this study, an altered regulation of Prox1-gene-expression is described in pathological processes of the liver.

## Methods

### Materials

Chemicals were purchased from Merck (Darmstadt, Germany), Applichem (Darmstadt, Germany) or Sigma (Steinheim, Germany). Materials for biochemical and immunohistochemical methods are listed in the appropriate sections.

### Liver Specimens

54 liver surgical specimens (16 peritumoral liver samples (cirrhotic and non-cirrhotic), 11 cirrhotic liver samples (without tumor), 19 HCC and 8 CCC) were used. 4 normal liver specimens were received from internal tissue bank archives. Informed consent was obtained from each patient included in the study and the study protocol conforms to the ethical guidelines of the 1975 Declaration of Helsinki as reflected in *a priori *approval by the institution's human local ethics committee.

### Cell lines, culture conditions

Hepatocellular carcinoma cell lines: HepG2 (purchased from the American Tissue Culture Collection (ATCC, Heidelberg, Germany)), HuH7 [14] and Hep3B (ATCC), were cultured conventionally in Dulbecco's MEM (Biochrom, Berlin, Germany) supplemented with 4.5 g/L glucose, antibiotics, 2 mM L-glutamine and 10% Fetal Calf Serum (PAA, Pasching, Austria). SK-ChA1, Mz-ChA1 and Mz-ChA2 biliary adenocarcinoma cell lines were received from Prof. A. Knuth (University Hospital, Zurich, Switzerland) [15] and were cultured in Dulbecco's MEM containing 4.5 g/L glucose and 3.7 g/L NaHCO_3 _supplemented with antibiotics, 2 mM L-glutamine, 1 mM Na-Pyruvate and 10% Fetal Calf Serum (PAA, Pasching, Austria).

### RNA isolation, northern blot analysis, real time RT-PCR

RNA was isolated with Trizol (Invitrogen, Carlsbad, CA, USA) from snap-frozen human liver specimens, cultured hepatoma and biliary adenocarcinoma cells, according to the instructions of the manufacturer. For northern blot analysis total RNA was separated by agarose gel electrophoresis, transferred onto a nylon membrane and hybridized with specific α^32^P-dCTP labeled (Amersham, Buckinghamshire, UK) cDNA probes (which were labeled with nick translation or random priming). Human Prox1 specific cDNA probe was kindly provided by Dr. SI. Tomarev [16], the 18S specific probe was synthesized using primers described elsewhere [17].

**Figure 1:**
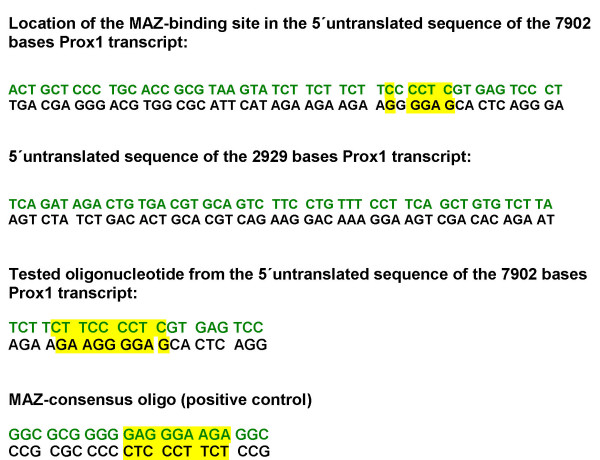
**Double-stranded oligonucleotide sequences designed for EMSA**. 5'untranslated sequences (5'-UTR) of the 7902 bases and of the 2929 bases transcript isoforms: green sequences are sense, black sequences are antisense. The MAZ-binding site was labelled in yellow. MAZ-binding site was not detected in the 5'-UTR of the "Prox1-2929"-transcript. MAZ-binding-site from data bank: CAC**GAGG**G**G**AAG; in the 5'-UTR of the Prox1-7902-transcript the binding site is lying in reverse position. In this figure the double-stranded oligonucleotide sequences are described, which were used in this study. (Literature reference: [23]; database reference: Ensembl Gene Report, OTTHUMG00000036946).

Reverse transcription of mRNA samples was performed with Mouse-Mammary Leukemia Virus Reverse-Transcriptase (Invitrogen), using random hexamers, according to the instructions of the manufacturer. Real-time RT-PCR analysis of cDNA- transcripts was performed with the Abi Prism Sequence Detection System 7000 of Applied Biosystems (Foster City, CA, USA) following the manufacturer's instructions, using Sybr-Green reaction master mix (Invitrogen) and the primers summarized in Table 1. RT-PCR for detection of Prox1 mRNA isoforms using exon- and isoform- specific primers was performed with the Go-Taq DNA Polymerase Master Mix (Promega, WI, USA) in a Perkin-Elmer Cycler (Waltham, MA, USA), using primers described in Fig. 1, and Additional file 1. Primers were designed based on sequence information provided by the Vertebrate Genome Annotation [VEGA] database (Cambridge, UK). Gene specific primers for 18S rRNA, Glyceraldehyde-3-phosphate dehydrogenase (GAPDH), beta-actin and beta-2-macroglobulin were designed as described previously [17]. Primers were synthesized by MWG Biotech (Ebersberg, Germany) or by Invitrogen. The comparative Ct method was used for analysis: the quantity of the PCR products was determined based on the threshold PCR cycle-values, and it was normalized with the quantities of the endogenous control PCR product, samples were analysed in triplets [17,18]. The normalised expression levels in all samples were related to the average normalised expression level in the normal liver, as a reference. Statistical significance was tested with Mann-Whitney "U"-test and one-way ANOVA. Several PCR products were sequenced by Seqlab (Göttingen, Germany), and the sequences were aligned by BLAST analysis (National Library of Medicine, Bethesda, MD, USA).

**Table 1:** Summary of the primers designed and used in the study.

**Gene**	**Forward primer**	**Reverse Primer**	**Amplicon length**	**mRNA location**
***Human Prox1 (Exon 3–4)***	5'-GCT CCA ATA TGC TGA AGA CC-3'	5'-ATC GTT GAT GGC TTG ACG TG-3'	120 bp	2399–2519
***Human HNF4α***	5'-GGG GTA CCC ATG GCC GAC TAC AGT G-3'	5'-GGG GTA CCG CGC TGA CAC CCA GGC TG-3'	140 bp	122 – 262
***Human albumin***	5'-CTG TGG GGC ACT TTT GAA AT-3'	5'-AAC ATC CCC TTC ACA GCA TC-3'	154 bp	698 – 851
***Human CREB***	5'-GAC ACC AGC TGG AGA AGG AC-3'	5'-CTT CTT GGT GTC CCA TGC TT-3'	198 bp	500 – 698
***Human AP-4***	5'-GTG CCC TCT TTG CAA CAT TT-3'	5'-CTG CCT TGC TGA GCT TCT CT-3'	229 bp	289 – 517
***Human N-myc***	5'-GAC TGT AGC CAT CCG AGG AC-3'	5'-GTT TTA ATA CCG GGG GTG CT-3'	161 bp	92 – 253
***Human MAZ***	5'-TAC CAC CTG AAC CGA CAC AA-3'	5'-GCT GCC TCA CAT TTC TCA CA-3'	247 bp	1040 – 1287

4 endogenous controls were tested whether they could be valuable for the normalisation of the *Prox1 *expression levels: the average expression of *beta-actin, beta-2-macroglobulin *and *glycerinaldehyde-3-phosphate dehydrogenase (GAPDH) *genes in HCC, CCC and cirrhotic liver samples were 1.65 – 2.06 fold increased compared to normal liver. This increase was significant by Mann-Whitney "U"-test. Meanwhile, 18S rRNA quantities did not show significant differences among the examined liver samples. Therefore, this was used for normalisation of Prox1 and other mRNA quantities.

### Immunostaining of cryosections

Liver sections of 5 μm thickness were cut with a cryostat (Reichert Jung, Wetzlar, Germany) air-dried for 10 minutes and used for immunohistochemical stainings after 10 minutes of acetone fixation at room temperature [10]. The following primary antibodies were used in the study: *rabbit polyclonal*: anti-Prox1 (1:200; Reliatech, Braunschweig, Germany), anti-Lyve-1 (1:500, Reliatech) and anti-Podoplanin (1:500, Reliatech).*Mouse monoclonal*: anti-Cytokeratin (CK) 7 (1:50, DAKO), anti-Cytokeratin (CK) 19 (1:100; Novocastra, Newcastle upon Tyne, England), anti-HepPar1 (clone: OCH1E5; 1:50; DAKO) and anti OV-6 (1:20; R&D Systems, Minneapolis, MN, USA). The rabbit polyclonal antibodies were detected with an Alexa-555-conjugated goat-anti-rabbit secondary antibody (Molecular Probes, Leiden, Netherlands). Mouse monoclonal antibodies were visualized with an Alexa-488-conjugated secondary anti-mouse antibody (Molecular Probes), diluted 1:200 in PBS. Sections were counter-stained with DAPI (Molecular Probes) and observed with an epifluorescence microscope (Axiovert 200 M, Zeiss, Jena, Germany). Negative control immunostainings were performed by omission of the primary antibody, by usage of a non-immune serum and by usage of isotype-matching control immunoglobulins.

### Isolation of cell nuclear extracts, western blot analysis

Cell nuclear proteins were extracted from cultured cells, and also from human liver tissue, as described before [19,20]. Ten microgram protein-containing nuclear extract samples were processed by sodium dodecyl sulfate polyacrylamide gel electrophoresis (SDS-PAGE) under reducing conditions [21]; followed by electroblotting [22]. The detection was performed according to the ECL protocol (Amersham, Buckinghamshire, England). Primary rabbit polyclonal antibody against Prox1 (Reliatech) was used at 5 μg/ml and primary monoclonal antibody against HNF4-alpha (Santa Cruz Biotech, Sanata Cruz, CA, USA) was used at 4.8 μg/ml concentration.

### Electrophoretic mobility shift assay (EMSA)

Double-stranded Myc-associated zinc finger protein (MAZ)-binding consensus- and control- oligonucleotides were designed based on a previous report [23]. Double-stranded oligonucleotides (described on Fig. 1; [23]) were labelled with [^γ-32^P]ATP (Amersham) using T4 polynucleotide kinase (Promega, Mannheim, Germany). Binding reactions were performed in a volume of 20 μl, comprising 10 μl of 2×-binding buffer (40 mM Hepes, 50 mM NaCl, 1 mM EDTA, 1 mM dithiothreitol and 0.5 mM PMSF, pH 7.5,) containing 5 μg of nuclear proteins, 300 ng of polydI-dC, 5 fmol ^32^P-end-labeled double-stranded oligonucleotide (8000 c.p.m.) [20]. For competition experiments a 100-fold molar excess of unlabeled double-stranded oligonucleotides was included in the DNA binding reaction [20,23]. Incubation was carried out at room temperature for 30 min [20], for supershift analysis at 4°C for overnight, using 2 μl of goat polyclonal anti-MAZ antibody (Santa Cruz); 3 μl loading buffer (50% glycerol, 1 mg/ml BSA) was added prior to loading on a 4% non-denaturing gel in 0.5× TBE buffer. Electrophoresis was performed at 170 V (in a Bio-Rad Protean II chamber (Bio-Rad, München, Germany)) for 110 min at 4°C. The gel was dried and autoradiographed [20].

### RNA silencing

The functional role of MAZ in the regulation of *prox1*-gene-expression was analyzed by transfection of HepG2 cells with pre-designed MAZ-specific siRNA reagents (siGENOME ON-TARGETplus SMARTpool duplex (Dharmacon-Perbio, Bonn, Germany) according to the instructions of the provider. As negative controls: Mock-transfection (no siRNA), and non-specific control siRNA (Cyclophilin B pool) were used. As positive control for the transfection siGLO Red Transfection Indicator was applied (Dharmacon-Perbio). 48 hours after the transfection (following the intructions of the provider) total RNA was isolated from the transfected cells, it was reverse transcribed, and analyzed by real time PCR using human Prox1 and MAZ-specific primers described in Table 1.

## Results

### Analysis of Prox1 immunohistochemical localization in normal human liver, in liver cirrhosis, in HCC and in CCC

**Figure 2:**
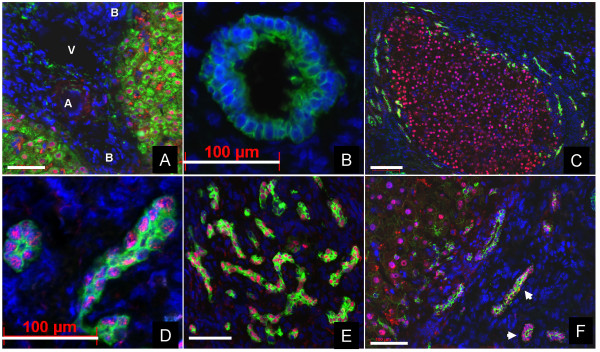
**Immunohistochemical analysis of Prox1 in normal human liver, in cirrhotic livers and in hepatocellular carcinoma**. ***A***) Double immunofluorescent labeling of HepPar1 (green), Prox1 (red) and cell nuclear counterstaining with DAPI (blue) in normal human liver shown combined. A: Arteria, B: Bile duct, V: Vena. Bars represent 100 μm. ***B***) Increased magnification of a bile duct within a normal human liver. Double immunostaining was performed with anti-CK7 (green) and anti-Prox1 (red). The blue staining with DAPI represents the nuclei. Bars represent 100 μm. ***C***) Immunohistochemical staining of a regenerative nodule in a cirrhotic liver with anti-CK7 (green), anti-Prox1 (red) antibodies and cell nuclear counterstaining with DAPI (blue), shown combined. Bars represent 100 μm. ***D***) Double immunostaining of CK7 (green) and Prox1 (red) in ductular cells within a fibrotic septum of a cirrhotic liver, cell nuclear counterstaining with DAPI (blue) shown combined. Bars represent 100 μm. E) Double immunostaining of CK19 (green) and Prox1 (red), cell nuclear counterstaining with DAPI in cells with a ductular phenotype within a cirrhotic liver shown combined. Bars represent 100 μm. ***F***) Immunohistochemical reactions of OV-6 (green) and anti-Prox1 (red) antibodies in HCC and the fibrous septa. The blue staining with DAPI represents the nuclei. Prox1^+ ^hepatocytes within the neoplastic nodule are surrounded by OV-6^+ ^ductular cells. Note that OV-6^+ ^cells are simultaneously Prox1^+ ^(white arrows). Bars represent 100 μm.

In normal human livers, Prox1-antigen was found in the nuclei of HepPar1^+ ^hepatocytes (Fig. 2A). Prox1 immunohistochemical reaction showed a homogeneous distribution in the liver parenchyma along with the HepPar1-staining, whereas CK-7^+ ^bile ducts were negative (Fig. 2A–B).

Hepatocytes within the so-called "regenerative nodules" of cirrhotic livers showed a strong Prox-1^+ ^immunohistochemical reaction (Fig. 2C). However, Prox1^+ ^cells with a ductular morphology were detected around regenerative nodules and within the fibrotic septa. Surprisingly, positive immunofluorescent reactions with antibodies described as biliary-type cytokeratins (CK) [24-28], i.e. CK-7, CK-19 and OV-6 were detected in the cytoplasm of these cells (Fig. 2C–F). Negative staining for HepPar1 revealed that these cells are not hepatocytes (data not shown). It was previously described that Prox1 is expressed in lymph endothelial cells. For this reason we applied markers of lymph epithelial cells in order to exclude reactions with lymph endothelium (LYVE-1 and podoplanin) [29]: these markers did not show co-localisation with Prox1 (data not shown).

**Figure 3:**
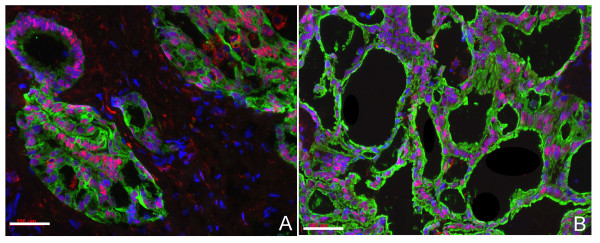
**Double immunostaining of Prox1 (red) with OV-6 or CK19 (green) in intrahepatic cholangiocellular carcinoma**. (***A***) OV-6; (***B***) CK19 show duct-like glandular or acinar structures localised within a dense fibrous stroma. Note that the cells are positive for OV-6 or CK19 and for Prox1. The blue colour staining with DAPI represents the nuclei. Bars represent 100 μm.

In HCC, cells within the neoplastic nodule showed Prox1^+ ^nuclei (Fig. 2F), and similarly to the cirrhotic liver, double Prox1^+^/OV-6^+ ^cells were detected in the fibrotic septa surrounding the neoplastic nodules. In CCC, ductular cells with remarkable Prox1-positivity were detected (Fig. 3A–B). These cells strongly reacted with anti-OV-6 (Fig. 3A) or with anti-CK19 (Fig. 3B), but not with HepPar1 (data not shown) and displayed a cuboid epithelial morphology.

### Analysis of *prox1*-gene-expression in HCC and biliary adenocarcinoma cell lines, in normal-, cirrhotic- liver, in HCC and CCC tissues

**Figure 4:**
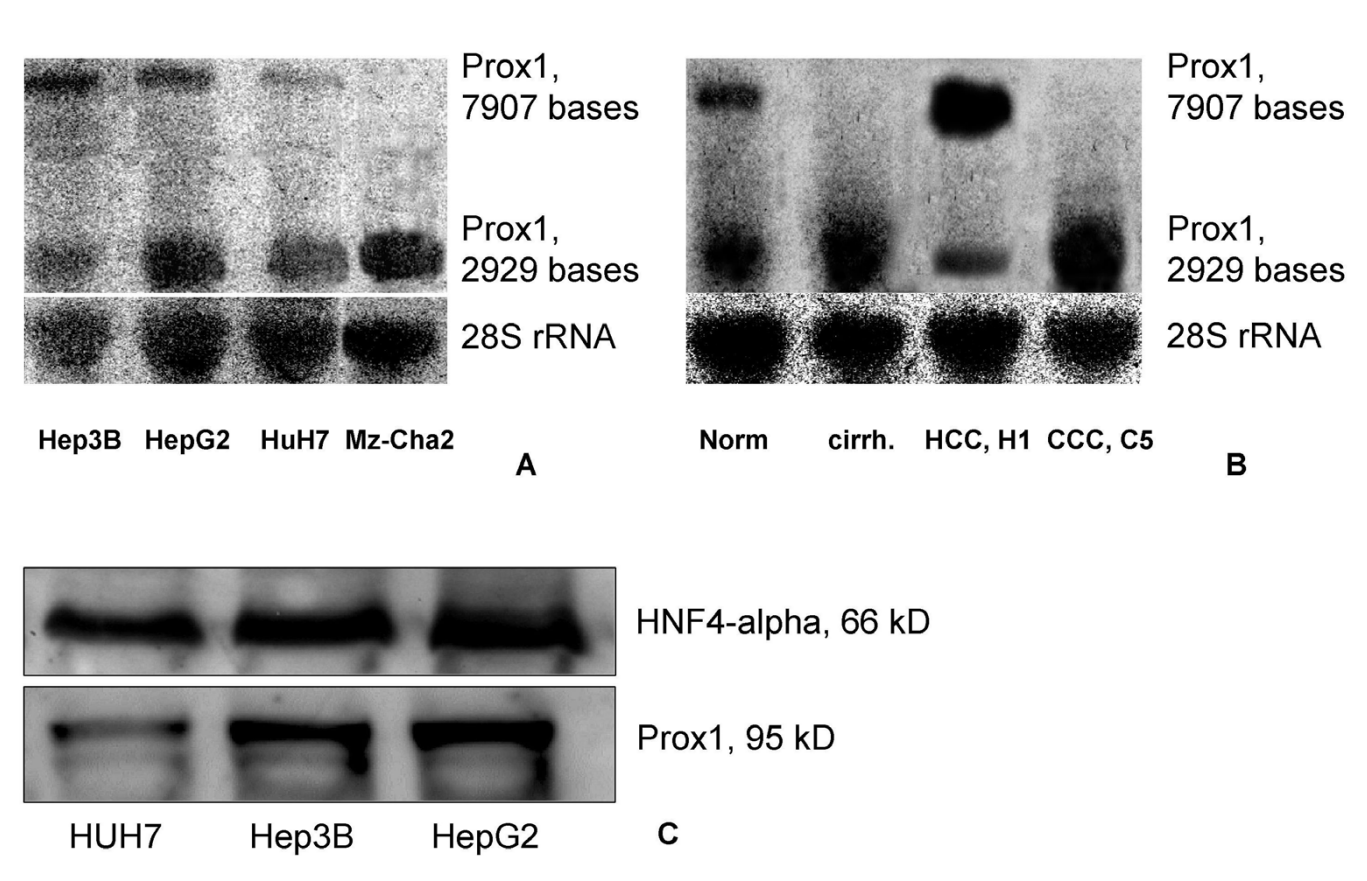
**Northern and western blot of Prox1, in normal liver, in liver cirrhosis in HCC and in CCC**. (***A***) Prox1 mRNA and 28s rRNA analysis on a northern blot of HCC cell lines (Hep3B, HepG2 and HuH7), and in a biliary adenocarcinoma cell line (Mz-Cha2). (***B***) Prox1 mRNA and 28s rRNA on a northern blot of human liver samples (normal liver, liver cirrhosis, HCC and CCC). Individual samples are labelled as on Figure 5.(***C***) Prox1 and HNF4-alpha western blot in HCC cell lines.

**Figure 5:**
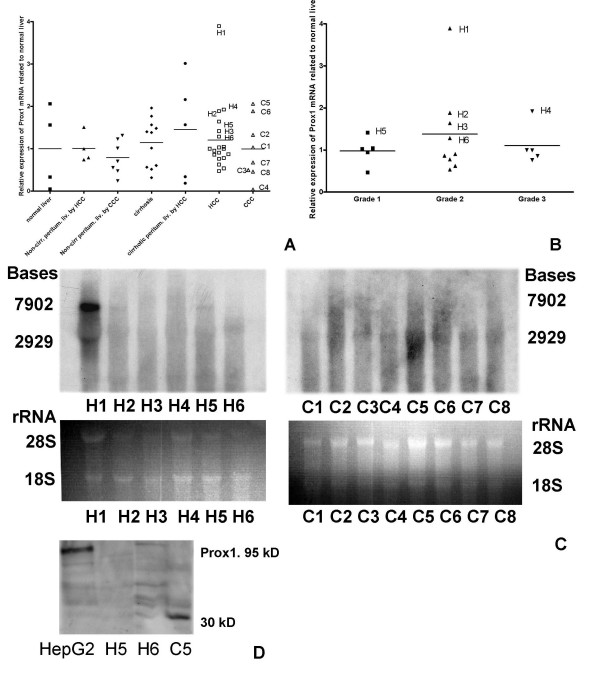
**Quantitative analysis of Prox1 mRNA expression in normal human liver, in peritumoral livers, in liver cirrhosis in HCC and in CCC**. (***A***). Steady-state levels of Prox1 mRNA compared to the *average expression *of normal liver, which is displayed as 1. (***B***) Prox1 mRNA expression levels (normalised with the endogenous control) related to the average expression of normal liver compared with grading. (**C**) Northern blot analysis of Prox1 mRNA expression of individual HCC (left panel) and CCC (right panel) samples (upper images). The gel photos of total RNA are shown as loading references (lower images). The same individual samples are also marked on the graphs of **A **and **B**. (**D**) Western blot analysis of Prox1 protein in nuclear extracts of some individual samples of **A-C, **nuclear extract of HepG2 was used as a positive control.

Using northern blot hybridisation more mRNA isoforms of Prox1 were found. Two of these isoforms were expressed in normal human liver, liver cirrhosis, in HCC and in CCC (Fig. 4B; Fig. 5C). The shorter isoform dominated in cirrhotic liver samples and in CCC, while the longer isoform was more abundant in HCC (Fig. 4B). The existence of other isoforms could not be excluded, but they were not clearly determined by hybridisation and sequencing in this study.

Human HCC cell lines (HepG2, HuH7 and Hep3B) showed high Prox1 expression, which was detected both at mRNA and protein levels (Fig. 4A,C). In all of the three cell lines both of the mRNA isoforms were detected, the quantitative relationship between those isoforms was different, i. e. HuH7 showed mainly the shorter, while Hep3B contained more of the longer isoform than of the short one (Fig. 4A). All of the three cell lines contained the same 95 kD Prox1 protein in their cell nuclear extract, HuH7 showed less Prox1-protein than HepG2 and Hep3B (Fig. 4C). No significant detection of Prox1-protein was found in the cytoplasmatic-extracts of these cells (not shown). These data imply the synthesis of a single protein from more mRNA isoforms.

At mRNA level, high Prox1-expression was detected in the biliary adenocarcinoma cell line MZ-Cha2 (Fig. 4A) as described before [13]. Interestingly, only the shorter form of Prox1 was expressed in MZ-Cha2 (Fig. 4A).

Prox1 mRNA expression was quantified by real-time RT-PCR using primers designed for a boundary sequence of exons 3–4 (Table 1), at similar location as it was described previously [12]. There was no significant difference in the average Prox1 mRNA expression level in normal liver, in non-cirrhotic or cirrhotic peritumoral liver, in liver cirrhosis, in HCC and in CCC compared to normal human liver, or compared with each other (p = 0.7932, with One-way ANOVA, Figure 5A, Table 2). A striking feature of Prox1 mRNA expression in all samples was a high variance (Fig. 5A). In liver cirrhosis and HCC *several samples *showed lower Prox1 mRNA expression compared to the *average *of the normal liver. Nevertheless, *some samples *of HCC and cirrhotic liver showed a higher Prox1 mRNA expression than the average of the normal liver (Fig. 5A). For more appropriate analysis individual investigation of some samples was required. More samples are shown individually on Fig. 5. Notably, one HCC sample showed an unexpected high Prox1-mRNA-expression, which was labelled with H1.

**Table 2:** Change in Prox1-mRNA-expression compared with the average of normal liver, in all analysed samples.

	**Upregulated % (n)**	**Not changed % (n)**	**Downregulated % (n)**
**Normal liver**	50 % (2)	-	50 % (2)
**Cirrhosis**	55.54 % (6)	9.09 % (1)	36.36 % (4)
**HCC**	31.57 % (6)	15.78 % (3)	52.36 % (10)
**Peritumoral (HCC), non-cirrhotic liver**	50 % (2)	25 % (1)	25 % (1)
**Peritumoral (HCC), cirrhotic liver**	60 % (3)	-	40 % (2)
**CCC**	37.5 % (3)	12.5 % (1)	50 % (4)
**Peritumoral (CCC), non-cirrhotic liver**	28.57 % (2)	14.28 % (1)	57.17 % (4)

Comparing Prox1 mRNA expression with differentiation grade in HCC, no significant difference could be found (p = 0.693, with One-way ANOVA, Fig. 5B).

In addition, correlation between albumin and Prox1 mRNA expression was analysed. Although, Prox1 expression levels significantly correlated with albumin levels in liver cirrhosis (p = 0.04, by correlation analysis, r^2 ^= 0.2717), no correlation could be detected in HCC (not shown).

The HCC samples, which showed higher mRNA expression than the average of normal liver were also analysed with northern blot (Fig. 5C, left panel). This method was the only way to demonstrate the Prox1-isoforms. Similarly to the real-time PCR data, the northern blot also showed variability of the Prox1-mRNA-expression in HCC. In samples H2 and H5 the dominance of the "Prox1-7902" isoform was detected. It is worthy to note the increased expression in H1 compared to the other samples.

All CCC samples were analysed by northern blot. In C1, C4–5 and C7–8 the dominance of the "Prox1-2929" isoform was detected (Fig. 5C, right panel).

Compared to the HCC cell lines, the cell nuclear extracts of HCC and CCC samples showed lower protein expression, and protein degradation products (Fig. 5D). Unfortunately, the H1 sample was not available any more for protein analysis.

Using primers described in Table 1 and in Additional file 1, mutation analysis was performed in all cDNA samples used in the study. Interestingly, the H1 sample (Fig. 5), with unexpectedly high Prox1 expression contained a point-mutation, which was detected using the primers described in Additional file 1. This mutation was located in the prospero domain, at the C-terminus after the homeodomain. The mutation changed Lys to Glu. In addition, the analysed sequence contained more insertions and deletions in the non-translated part (Additional file 1).

### Databank evidence for more Prox1-mRNA-isoforms

The northern blot results were compared with Genebank data. Based on recent database entries, different human Prox1 mRNA isoforms are described. In the Vertebrate Genome Annotation Database ("Human And Vertebrate Analysis aNd Annotation", HAVANA) of the Sanger Institute (Cambridge, UK) two human Prox1 mRNAs are available, one consists of 7907, the other of 2929 bases.

**Figure 6:**
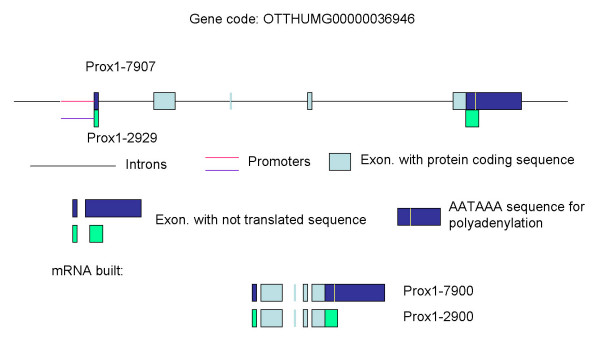
**Prox1 mRNA isoforms**. Scheme of the exon-intron construction of Prox1-mRNA-isoforms.

Isoform-specific primer sets were designed for further analysis of the two Prox1 mRNA isoforms. The primers targeted the different exons of the two isoforms (Fig. 6). Both, the 2929- and 7907-bases variants include the same protein coding sequence. A major part of the last exon in the 7907-bases-variant is not translated. Primers were designed to the first exon of the 7907-bases variant, where the forward primer reacted with the first part of the untranslated exon-1, and the reverse primer was located in exon-2 (Additional file 1). Primers were designed to a similar position of exon 1–2 of "Prox1-2929". The forward-primer reacted with an untranslated region of exon-1 of "Prox1-2929"mRNA, while the reverse primer was the same as by the 7907-base variant (Additional file 1). It is clear from the Additional file 1 that, the first exons of the two isoforms of Prox1 contain different sequences. Nevertheless, these different regions are not translated.

Further analysis was performed on the other side of the Prox1-mRNA-sequence, regarding the last exon. The "Prox1-end" primer set amplified a sequence starting at Exon 4, and finishing at Exon 5 of "Prox1-7907". The forward primer included base pairs before and after Intron 4–5 to pick up transcribed gene only and to avoid picking up genomic DNA. The reverse primer included an AATAAA sequence [in mRNA AAUAAA], which is the signal for polyadenylation (Additional file 1) [30,31].

In the analysed liver samples the mRNA isoforms, which were described in the databank, were confirmed by RT-PCR-amplification and subsequent gel-electrophoresis, and also by sequencing.

In the majority of the HCC samples the specific primers for the last exon of the 7907 bases isoform, amplified the 1166-bases PCR product (not shown). In CCC samples the same primers amplified a PCR product with less efficiency, showing less or no product in several cases, or unspecific bands (not shown). In both HCC and CCC the first exons of both of the 7907 and of the 2929 bases isoforms were detected (not shown).

### Possible regulation of the *prox1*-gene-expression

"In silico" analyses of the 5'-flanking sequences of Prox1 isoforms, revealed binding sites for different transcription factors.

**Figure 7:**
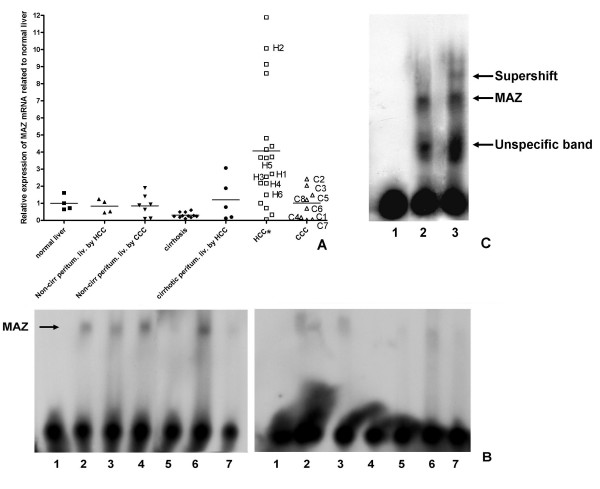
**mRNA expression of MAZ in normal human liver, in peritumoral liver, in liver cirrhosis, in HCC and in CCC, MAZ-binding to the Prox1 promoter**. **(A) **mRNA expression levels of MAZ in normal human liver, in non-cirrhotic peritumoral liver, in liver cirrhosis, in cirrhotic peritumoral liver, in HCC and in CCC compared to the average expression of normal liver, which is displayed as 1. HCC displayed a significant upregulation, marked with asterisk. The same individual samples are marked as the ones on Fig. 5. **(B) **EMSA reaction of cell nuclear extracts of HepG2 (2), Hep3B (3) and Mz-Cha2 (4), cell lines, and of nuclear extracts of HCC (5–6) and CCC samples (7) incubated for 30 minutes with the MAZ-consensus binding oligo-containing 5'-flanking sequence of the "Prox1-7902"-transcript (Fig. 1) (left panel). When the nuclear extracts were reacted with the same radiolabelled consensus double-stranded oligo in the presence of a 100-fold excess of an unlabeled MAZ-consensus oligo (published in ref. [23]) the intensity of mobility shift significantly decreased (right panel); sample 1: negative control, without nuclear extract. **(C) **EMSA reaction of cell nuclear extracts of Hep3B (2–3) cells incubated for *overnight *with the MAZ-consensus binding oligo-containing 5'-flanking sequence of the "Prox1-7902"-transcript, without (2) and with (3) anti-MAZ antibody; sample 1: negative control, without nuclear extract. The strong unspecific band might represent other transcription factors binding to the same sequence. In the presence of the anti-MAZ antibody a supershift band was observed (lane 3).

In the 5'-flanking sequence of the "7907-Prox1" among others the consensus binding sequences of N-Myc and Myc-associated Zinc-Finger (MAZ) were identified. Interestingly, in contrast to N-Myc, MAZ-expression was detected in all analysed liver samples (Fig. 7A, Table 3), and cell lines (Table 4). While normal and peritumoral non-cirrhotic liver, cirrhosis and CCC showed a similar MAZ expression (p = 0.3112, by one-way ANOVA), significantly increased expression could be found in HCC (p = 10^-4^, by one-way ANOVA), (Fig. 7A, Table 3). MAZ-gene-expression was upregulated in 78.94 % of the HCC samples, compared to the average of the normal liver (Table 3). In the 5'-flanking sequence of the "2929-Prox1" binding sites for several transcription factors were found (i. e. cAMP-responsive Element Binding Protein (CREB) and Activator protein 4 (AP-4)). Although, the mRNA expression of these genes was recognised, no significant change was seen in the expression in liver cirrhosis, HCC and CCC compared with normal human liver (p = 0.38; 0.144 for AP-4 and CREB, respectively, with one-way ANOVA), (not shown).

**Table 3:** Change in MAZ-mRNA-expression compared with the average of normal liver, in all analysed samples.

	**Upregulated % (n)**	**Not changed % (n)**	**Downregulated % (n)**
**Normal liver**	25 % (1)	25 % (1)	50 % (2)
**Cirrhosis**	-	-	100 % (11)
**HCC**	78.94 % (15)	5.26 % (1)	15.78 % (3)
**Peritumoral (HCC), non-cirrhotic liver**		50 % (2)	50 % (2)
**Peritumoral (HCC), cirrhotic liver**	40 % (2)	-	60 % (3)
**CCC**	50 % (4)	-	50 % (4)
**Peritumoral (CCC), non-cirrhotic liver**	28.57 % (2)	42.85 % (3)	28.57 % (2)

**Table 4:** Typical Ct-Values of the real-time RT-PCR for MAZ-expression in HCC and CCC cell lines.

HepG2	24.32
HuH7	24.64
Hep3B	23.34
SK-Cha1	25.17
MZ-Cha1	24.31
MZ-Cha2	24.05

Using EMSA, a significant binding was found to the MAZ-consensus site-containing the 5'-flanking sequences of the "Prox1-7902" transcript, in the cell nuclear extracts of HCC cell lines, of Mz-Cha2 cell line and of a HCC tumor sample. This binding capacity was suppressed by the 100-fold excess of the MAZ-consensus oligo [23] (Fig. 7B). The presence of the MAZ transcription factor in the cell nuclear extract of a HCC cell line (Hep3B) was further confirmed by a supershift assay, using a MAZ-specific goat polyclonal antibody (Fig. 7C).

Following siRNA transfection in HepG2 cells, the MAZ mRNA expression was reduced to 8.38% of the control, while transfection with siRNA for cyclophilin B did not cause suppression of MAZ mRNA. Interestingly, following siRNA-mediated inhibition of MAZ mRNA expression, the expression level of Prox1 was significantly reduced (p = 0.03, with Student's t-test) to 41.72 % of the control (Table 5). These data indicate the positive regulatory potential of MAZ in the Prox1-gene-expression of HCC.

**Table 5:** Relative expression of MAZ and Prox1 in HepG2 cells after Mock transfection, transfection with cyclophilin B siRNA (negative control) and with MAZ siRNA

**Sample**	**MAZ relative expression compared to Mock transfection**	**Prox1 relative expression compared to Mock transfection**
**Mock transfection**	1 ± 0.039	1 ± 0.15
**Cyclophilin B si RNA transfection**	1.602 ± 0.023	0.9 ± 0.06
**MAZ siRNA transfection**	0.084 ± 0.025	0.417 ± 0.16

## Discussion

Up to now the main function of Prox1 in the liver is not well known. Previously we found that, Prox1 is expressed in hepatocytes of normal and injured rat livers, whereas it is absent in biliary epithelial cells [9,10].

Furthermore, we have studied the effects of Prox1 on the transcriptional profile of met-murine hepatocytes (MMH). These immortalized murine hepatoblasts express numerous hepatoblast markers, but not Prox1. A stable transfection with Prox1 cDNA and transcriptome analyses, revealed an up-regulation of 22 genes and down-regulation of 232 genes. Many of these genes are involved in hepatocyte functions, suggesting that Prox1 is a multifunctional regulator of liver morphogenesis and of hepatocyte function [32]. Proliferation of MMH was not altered by stabile transfection with Prox1 [32].

Other researchers suggested a tumor suppressor function for Prox1 [12,13] in HCC and CCC, and recently also in other tumors [33].

In the current study we could detect a high variability of Prox1 mRNA expression among normal, cirrhotic human liver specimens, in HCC and in CCC. No significant relationship was found between Prox1 mRNA expression and differentiation grade.

The prox1 gene is located on the long arm of chromosome 1 (1q32), where a loss of heterozygosity in malignant lymphoma was observed [34]. This is an instable region of the genome. Accordingly, we found a point mutation in the prospero domain in one HCC sample with unexpected high Prox1 expression. This mutation might eliminate or alter the function of the *prox1 *gene [35]. Similarly to previously described mutations [35], this mutation also occurred at evolutionary conserved codons [36].

Our findings indicated that, apart from hepatocytes, Prox1-expression was also detectable in ductular cells within the fibrotic septa of cirrhotic livers and in the nuclei of intrahepatic cholangiocellular carcinoma cells. In order to identify Prox1^+ ^cells in the investigated human liver samples, lineage-specific markers were used. HepPar1 was described as a hepatocyte marker [24] and was also observed in liver cells with intermediate fate (hepatocyte/cholangiocyte) in a variety of acute and chronic human liver diseases [24,37-39]. CK7, CK19 and OV-6 biliary type cytokeratins have been described in "oval cells" as well [26,40]. OV-6 is a monoclonal antibody rose against cells isolated from 2-acetylaminofluorene-treated rat liver, which recognize the epitope on cytokeratins (CK) 19 and 14, and is a widely used "oval cell" marker [27,28,38,41].

Cells of morphology and immunophenotype intermediate between hepatocytes and cholangiocytes ("intermediate cells") were not recognized in normal liver tissue [42]. Surprisingly, ductular cells, which were positive for biliary epithelial cell markers (CK7, CK19, OV-6) [42] and located within the fibrotic septa and at the margin of regenerating nodules of cirrhotic livers, showed a positive-staining for Prox1 as well. Morphologically, these cells were different from hepatocytes and had features shared with biliary epithelial cells (small oval to cuboidal cells with round to oval nuclei). These findings support that Prox1^+ ^cells of CCC might descend from the biliary epithelial cell lineage, and Prox1 is newly expressed in those cells.

The current study provided data, which might take an insight into the regulation of Prox1 expression under pathological conditions. This study confirmed the existence of more mRNA isoforms, which was shown by Zinovieva *et al*., in 1996 [36]. In this study two isoforms were revealed by hybridisation and sequencing. In the previous report of Zinovieva *et al*. (1996) [36] an additional unclear hybridisation smear was observed between the long and the short transcript in the human embryonic lens samples. Similarly, on our Fig. 5C unclear hybridisation results were observed in more samples. These observations indicate the existence of more Prox1 transcripts, which were discovered more than 20 years ago. Identification of even more Prox1 mRNA transcript might be expected in the future. These isoforms might be regulated by different regulatory sequences, but the same sequences might influence more isoforms based on their position (OTTHUMG00000036946 in the VEGA database). The dominance of the 7907 bases form was found in more hepatocellular carcinoma samples, while the shorter isoform was more often dominant in CCC (Fig. 5C), and in the MZ-Cha2 biliary adenocarcinoma cell line. The induction of the shorter Prox1 mRNA isoform might be related with a neoexpression in a cell population with biliary phenotype in liver cirrhosis and in CCC.

The results of the current work suggest that in HCC the Myc-associated Zinc-finger protein (MAZ) might be involved in the expression Prox1. The expression of MAZ is significantly increased in HCC. It was demonstrated here that, the MAZ protein might directly bind to responsive elements included in the regulatory sequences of Prox1, and might contribute to the positive regulation of this gene (Fig 7, Table 5). MAZ was originally found to bind specifically to the wild-type ME1a1 sequence, a 16-base-pair nuclear factor binding site residing between the *c-myc *P1 and P2 transcription initiation sites, the MElal site facilitate initiation from the downstream start site of c-myc [43]. Northern blot analysis revealed that MAZ is expressed in human heart, brain, placenta, lung, liver, skeletal muscle, and pancreas [43]. It was shown that co-expression of c-myc and transforming growth factor (TGF)-alpha as transgenes in mouse liver results in major enhancement of neoplastic development [44]. C-myc contributes to hepatocarcinogenesis not only by regulating cell proliferation and death but more profoundly by modulating specific metabolic pathways [45]. Based on the current study, MAZ might be involved also in the regulation of Prox1 in HCC. Prox1 might influence several genes involved in hepatic metabolic functions [32].

The Prox1 isoforms not only differ by their 5'-flanking regions, but also by their end-sequence. In the 3'-non-translated area the unprocessed pre-mRNA contains a significant signal-motive, which functions as a signal for polyadenylation, recognised by *Cleavage and Polyadenylation Specificity*-*Factor *(CPSF) [30,31] Although alternative sequences also exist, the AAUAAA is the most wide-spread polyA signal. AAUAAA is detectable after the TGA stop codon in the "Prox1-7907", but not in the "Prox1-2929". This difference might be related with a higher stability of the "Prox1-7907", which dominates over the short isoform in HCC. Accordingly, MAZ might be also involved in transcriptional stabilisation, not only in initialisation of the transcription [43].

## Conclusion

In summary, the current study provided further evidence for the existence of different Prox1 mRNA isoforms in human liver, and recognised the dominance of a 7907-bases isoform in HCC, and the 2929-bases isoform in CCC. In some HCC cases the Prox1 mRNA expression was higher compared to the average of the normal liver, which in one case was related with mutation. At protein level the expression of the 95 kD protein was lower in liver tumors, or degradation was detected in the cell nuclear extracts. Human HCC cell lines contained high Prox1 expression both at protein and mRNA levels. The expression of the "Prox1-7907"-mRNA-isoform, and the high expression of Prox 1 in HCC cell lines might be associated with the increased expression of MAZ transcription factor, which is able to bind to the promoter of "Prox1-7907". A direct linear relationship between MAZ and Prox1 expressions could not be stated. Our histological analysis detected the neoexpression of Prox1-protein in ductular biliary cells of liver cirrhosis and of CCC.

Although our understanding is continuously increasing, there are still several puzzles and contradictions related to this homeobox gene in hepatic carcinogenesis, which should be solved in the future.

## Abbreviations

AFP-α-Fetoprotein, ANOVA-Analysis of Variance, AP-4-Activator protein 4, CCC-Cholangiocellular Carcinoma, CK-Cytokeratin, CPSF-Cleavage and Polyadenylation Specificity-Factor, CREB-cAMP-responsive Element Binding Protein, ED-Embryonic Day, EMSA-Electrophoretic Mobility Shift Assay, HAVANA-Human And Vertebrate Analysis aNd Annotation, HCC-Hepatocellular Carcinoma, HNF-Hepatic Nuclar Factor, MAZ-Myc-associated Zinc-finger protein, MEM-Minimum Essential Medium, Prox1-Prospero-related Homeobox 1, TGF-Transforming Growth Factor

## Competing interests

The author(s) declare that they have no competing interests.

## Authors' contributions

JD carried out the molecular genetic studies, the sequence alignment, designed primers, analysed sequences, performed northern blots, western blots, EMSA, siRNA transfections and analysis, performed the statistical analysis, drafted and revised the manuscript. TM carried out the immunoassays, data analysis, drafted and corrected the manuscript. FM participated in the molecular genetic studies, FH participated in the molecular genetic studies, in the sequence alignment, primer design and the statistical analysis. JW participated in the design and coordination and helped to draft and revise the manuscript. TL and LF participated in the data achievement, in analysis of clinical data, and in the correction of the manuscript. GR conceived and designed the study, participated in data analysis, discussed the results, directed the flow of the study, and corrected the drafts. All authors read and approved the final manuscript.

## Pre-publication history

The pre-publication history for this paper can be accessed here:



## Supplementary Material

Additional file 1 "Additional sequence data supporting the study". The data provided represent the position of the primers for amplification of the isoform-specific PCR-products and the localisation of a point mutation in sample H1. Click here for file
