# Supplementary material for: Altered regulation of Prox1-gene-expression in liver tumors
Source: BMC Cancer. 2008 Apr 9;8:92. doi: 10.1186/1471-2407-8-92 (PMC2359759; doi:10.1186/1471-2407-8-92)
Supplement: Additional file 1 — "Additional sequence data supporting the study". The data provided represent the position of the primers for amplification of the isoform-specific PCR-products and the localisation of a point mutation in sample H1. [file 1471-2407-8-92-S1.doc]

# Supplementary Figures


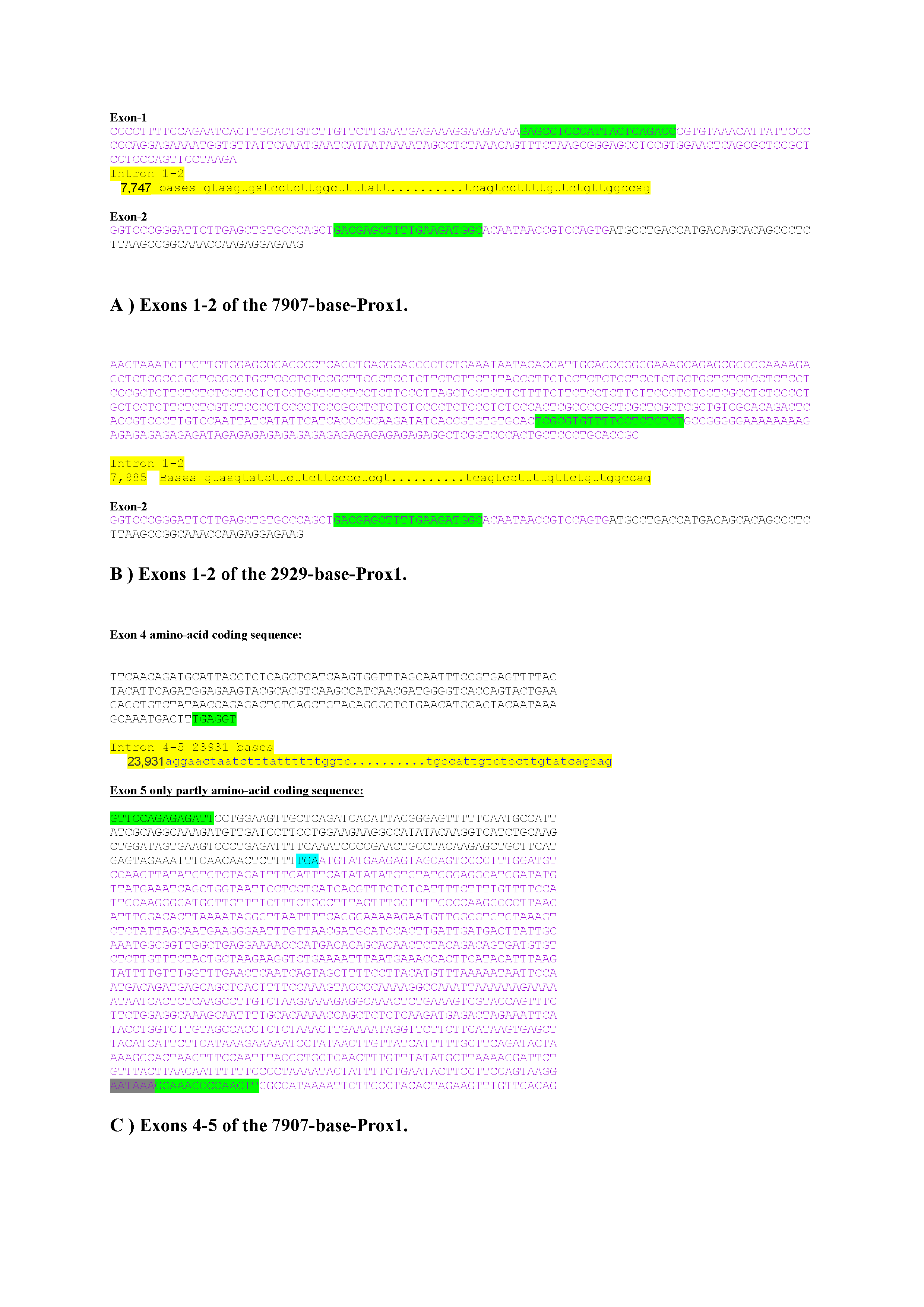


**Supplementary Figure 1.**

**Position of the primers for amplification of the isoform-specific PCR-products.**

Protein-coding sequence is written in grey, non-coding sequence in purple, intron sequence in small caption, primer-positions are labelled with green, stop codon (TGA) is labeled in blue, AATAAA signal for polyadenylation is labelled in grey. All sequences are written as DNA (cDNA, PCR-product) sequences. The primers in **Supplementary Figure 1C** were also used for mutation analysis and detected a mutation in sample H1 (Fig. 5).

**In wild type human Prox1 transcript (OTTHUMT00000089727)**

2281 ATAAAGCAAATGACTTTGAGGTGTTCCAGAGAGATTCCTGGAAGTTGCTCAGATCACATT

670 N--K--A--N--D--F--E--V--F--Q--R--D--S--W--K--L--L--R--S--H--

**In Sample H1**

2281 ATAAAGCAAATGACTTTGAGGTGTTCCAGAGAGATTCCTGGGAGTTGCTCAGATCACATT

670 N--K--A--N--D--F--E--V--F--Q--R--D--S--W--E--L--L--R--S--H--

**Supplementary Figure 2.**

**Localisation of a point mutation in sample H1 (Fig. 5).**

Interestingly, the H1 sample (Fig. 5), with unexpectedly high Prox1 expression contained a mutation, which was detected using the primers described in Supplemental Fig. 1 C. This mutation was located in the prospero domain, at the C-terminus after the homeodomain. The mutation changed Lys (K) to Glu (E). In addition, the analysed sequence contained more insertions and deletions in the non-translated part. The Homo sapiens prospero-related homeobox 1 (PROX1), transcript sequence was used from the VEGA database, with reference number: **OTTHUMT00000089727**.
